# A combination of circulating miRNAs for the early detection of ovarian cancer

**DOI:** 10.18632/oncotarget.20688

**Published:** 2017-09-06

**Authors:** Akira Yokoi, Yusuke Yoshioka, Akihiro Hirakawa, Yusuke Yamamoto, Mitsuya Ishikawa, Shun-ichi Ikeda, Tomoyasu Kato, Kaoru Niimi, Hiroaki Kajiyama, Fumitaka Kikkawa, Takahiro Ochiya

**Affiliations:** ^1^ Division of Molecular and Cellular Medicine, National Cancer Center Research Institute, Tokyo, Japan; ^2^ Department of Obstetrics and Gynecology, Nagoya University Graduate School of Medicine, Nagoya, Japan; ^3^ Statistical Analysis Section, Center for Advanced Medicine and Clinical Research, Nagoya University Graduate School of Medicine, Nagoya, Japan; ^4^ Department of Gynecology, National Cancer Center Hospital, Tokyo, Japan

**Keywords:** circulating microRNAs, ovarian cancer, exosomes, biomarkers, liquid biopsy

## Abstract

Ovarian cancer is the leading cause of gynecologic cancer mortality, due to the difficulty of early detection. Current screening methods lack sufficient accuracy, and it is still challenging to propose a new early detection method that improves patient outcomes with less-invasiveness. Although many studies have suggested the utility of circulating microRNAs in cancer detection, their potential for early detection remains elusive. Here, we develop novel predictive models using a combination of 8 circulating serum miRNAs. This method was able to successfully distinguish ovarian cancer patients from healthy controls (area under the curve, 0.97; sensitivity, 0.92; and specificity, 0.91) and early-stage ovarian cancer from patients with benign tumors (0.91, 0.86 and 0.83, respectively). This method also enables subtype classification in 4 types of epithelial ovarian cancer. Furthermore, it is found that most of the 8 miRNAs were packaged in extracellular vesicles, including exosomes, derived from ovarian cancer cells, and they were circulating in murine blood stream. The circulating miRNAs described in this study may serve as biomarkers for ovarian cancer patients. Early detection and subtype determination prior to surgery are crucial for clinicians to design an effective treatment strategy for each patient, as is the goal of precision medicine.

## INTRODUCTION

Every year, 240,000 women are diagnosed with ovarian cancer (OvCa) worldwide, and OvCa is the leading cause of gynecologic cancer mortality [1]. The dismal outcomes of OvCa are mainly due to late-stage diagnosis. Over 70% of patients are diagnosed at an advanced stage, and the overall 5-year survival for FIGO Stage III and IV OvCa is only 23% [2, 3]. However, the few patients who are fortunately diagnosed at FIGO stage l have a 5-year survival of over 90% [4]. To date, no gold standard screening method has been established for OvCa, and patients are typically detected through multimodal screening approaches incorporating CA-125 and ultrasound [5]. Serum biomarkers are attractive targets for disease screening because they can provide useful information without invasive procedures. Most recently, the results of a large-scale randomized controlled trial assessing the current optimal screening method, which includes CA-125 measurement and ultrasonography, found that the mortality reduction was not significant [6]. Thus, there is an urgent need to develop new strategies that can detect OvCa at an early stage as well as in asymptomatic women to improve patients’ outcomes.

MicroRNAs (miRNAs) are small noncoding RNAs that play various roles in physiology and disease development [7, 8]. Some miRNAs are secreted from cells and circulate stably in body fluids [9]. Recently, extracellular RNA (exRNA) has received a lot of attention as a new area of research, and the various forms of exRNA in body fluids, including miRNAs, represent potential next-generation biomarkers that are being investigated for clinical use [10].

The existence of OvCa-associated circulating miRNAs was reported within the past decade. Several studies identified serum miRNAs that were correlated with patient clinical status, and could predict prognosis and chemosensitivity [11–13]. Despite these interesting reports, progress in the development of reliable serum biomarkers for early detection remain very limited due to the small size of available research cohorts. In the present study, we performed miRNA sequencing to identify candidate miRNAs that could be useful in early detection of OvCa and subtype classification. We identified 8 miRNAs, which we validated by qRT-PCR, and we applied statistical cross-validation methods to a large research cohort to determine the optimal combination of miRNAs to incorporate in prediction models.

## RESULTS

### Identification of serum circulating miRNAs as potential ovarian cancer biomarkers

**Figure 1:**
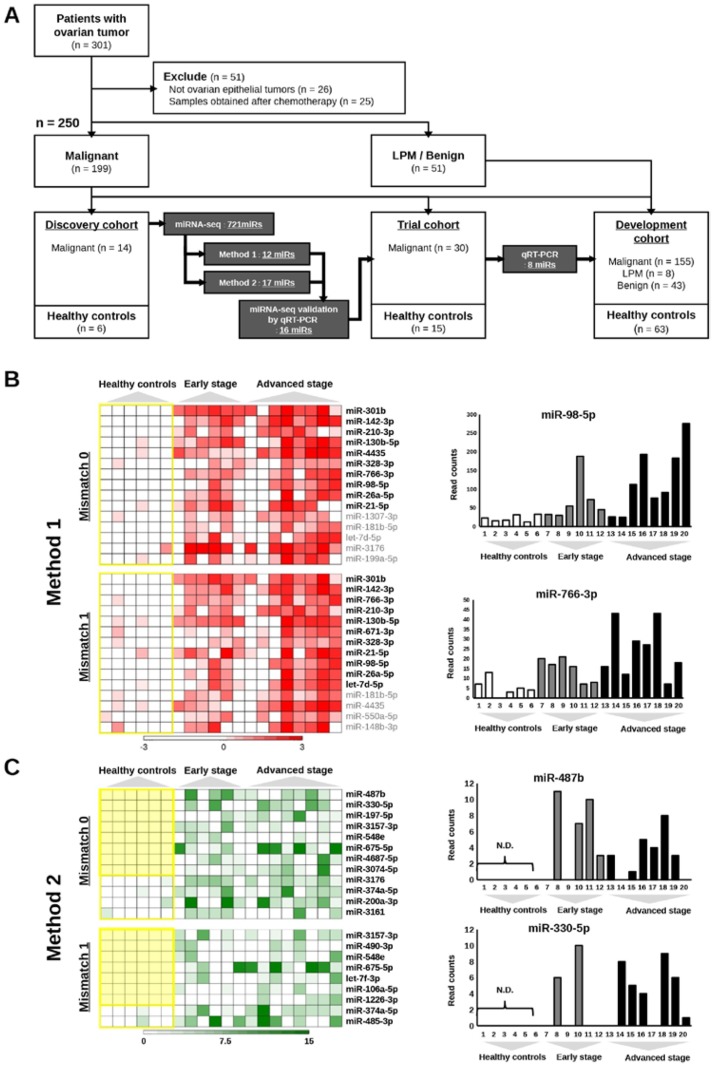
An overview of the process for selecting candidate miRNAs **(A)** Flowchart of the process for the development of biomarkers for early-stage ovarian cancer. A total of 301 serum samples were prepared from patients with ovarian tumors, and 250 samples were analyzed. Eighty-three samples from healthy controls were also prepared. Candidate miRNAs were selected based on the results of miRNA-seq, and further narrowed down using multiple validation steps. LPM: low potential of malignancy. **(B)** Results of selection from miRNA-seq using method 1. The heat maps show the distribution of read counts for miRNAs using the data with 0 or 1 mismatch allowed. The yellow line encloses results from healthy controls. The miRNAs shown in bold were selected. The bar charts on the right show the read counts of 2 miRNAs (miR-98 and miR-26a-5p) as examples. Read counts are on the vertical axis, and samples are on the horizontal axis. **(C)** Results of selection from miRNA-seq using method 2. The heat maps show the distribution of read counts for miRNAs using the data with 0 or 1 mismatch allowed. The yellow line encloses results from healthy controls. The miRNAs shown in bold were selected. The bar charts on the right show the read counts of 2 miRNAs (miR-487b and miR-330-5p) as examples. Read counts are on the vertical axis, and samples are on the horizontal axis.

**Figure 2:**
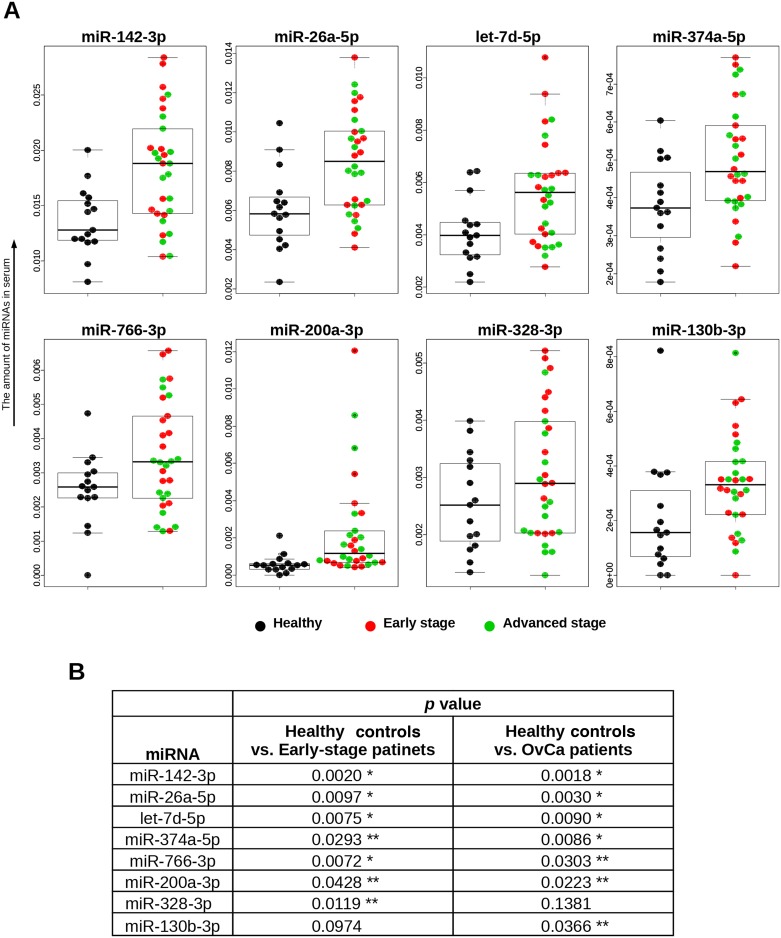
Distributions of 8 selected miRNAs in trial cohort **(A)** Serum levels of miRNAs. The dot plots are overlaid with box plots. The vertical axis shows 2^⊿Ct values, which were normalized to the values for cel-miR-39. Descriptions of the data points are shown below the graphs. **(B)** Statistical analyses. The Mann–Whitney *U*-test was used. ^*^p < 0.01, ^**^ p < 0.05.

An overview of the process for the identification of miRNAs highly predictive of OvCa is illustrated in Figure 1A. To identify candidate miRNAs, we performed global miRNA expression profiling (miRNA-seq) using total RNA extracted from 600 μL of serum from healthy controls (n = 6), patients with early-stage OvCa (stages I and II; n = 6) and patients with advanced-stage OvCa (stages III and IV; n = 8). The detailed summary of clinical information in the discovery cohort are presented in Supplementary Table 1. The quality control procedures for sequence data are described in Supplementary Table 2, and all the read count data are provided in Supplementary Tables 3-4. As shown in the heat map, a total of 721 miRNAs were successfully identified (Supplementary Figure 1). To establish a diagnostic model for the detection of OvCa, particularly early detection of stage I and II OvCa, we used two different criteria. The first set of criteria (method 1) identified miRNAs that exhibited statistically significant differences in expression between cancer patients and controls (Figure 1B), and the second set of criteria (method 2) identified miRNAs whose expression was detected only in cancer samples (i.e., read counts were almost 0 in healthy controls, as shown in Figure 1C). Because the optimal biomarker is one that would be detected only in the disease cohort and not in healthy patients, or *vice versa* (i.e., all-or-none expression), we used both sets of criteria. To select the maximum number of candidates from the sequencing data, our standard mapping parameters were set to allow up to 1 mismatch per miRNA sequence (Figure 1B and 1C). Over 40 miRNAs showed statistically significant difference in expression between cancer patients and healthy controls (p < 0.01). The top 10 miRNAs with the most significant differences in expression from both the 0-mismatch and the 1-mismatch mapping results were retained; given the overlap between both sets of results, a total of 12 miRNAs were selected in this step (Figure 1B). Similarly, we identified 17 miRNAs with statistically significant differences in expression (p < 0.05, cancer vs. healthy) that had read counts of 0 in over 5 healthy controls (Figure 1C). To test the reproducibility of these 29 miRNAs by qRT-PCR analysis, RNA was re-extracted from 200 μL of serum in the same sample set that was used for the initial miRNA-seq (Supplementary Table 1). As a result, 16 of the 29 miRNAs were validated, as they showed statistically significant differences in expression and prominent trends indicating that they are highly expressed only in the sera of cancer patients (Figure 1A and Supplementary Figure 2). Furthermore, the correlation data for the miRNA-seq read counts and the CT values of the qRT-PCR were provided in Supplementary Figure 3. In subsequent analyses, we used 45 serum samples as an independent trial set (Supplementary Table 5) and identified 8 miRNAs with statistically significant differences in expression that were able to predict the development of OvCa and early-stage OvCa (Figure 2A–2B). Although there were no miRNAs featuring an all-or-none expression pattern in the qRT-PCR analysis, 2 out of the 8 miRNAs (miR-200a-3p and miR-374a-5p) that were retained in this final step had been identified using method 2.

### Diagnostic performance of 8 miRNAs in the development set

**Figure 3:**
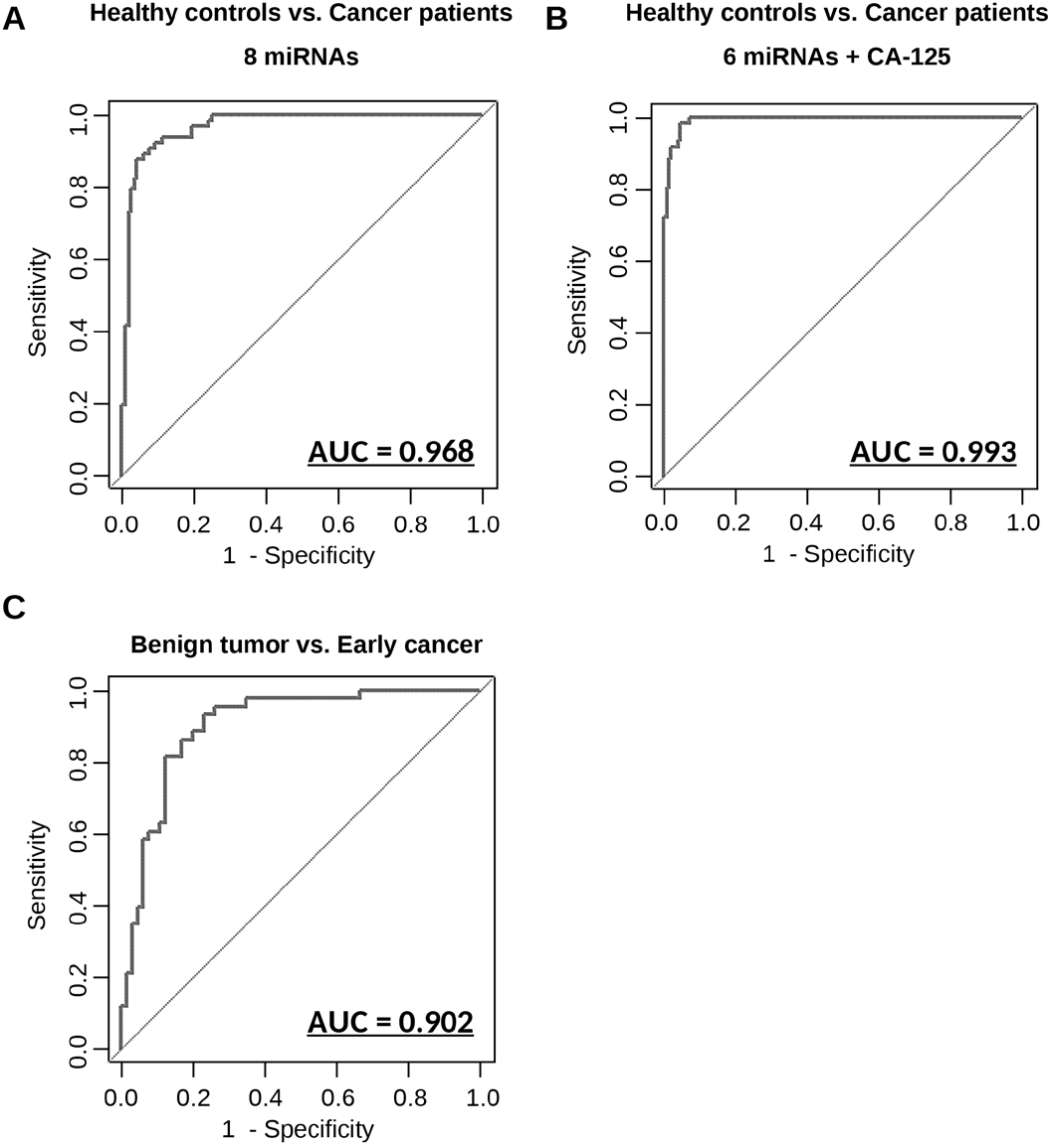
Diagnostic outcomes in each model for the prediction of ovarian cancer **(A)** ROC curve for identification of patients with ovarian cancer (N = 155) versus healthy controls (N = 63) using 8 miRNAs. **(B)** ROC curve for identification of patients with ovarian cancer (N = 155) versus healthy controls (N = 63) using 6 miRNAs and CA-125. **(C)** ROC curve for identification of early-stage patients with ovarian cancer (N = 65) versus patients with benign ovarian tumors (N = 43) using 7 miRNAs. The AUC values are shown on the graphs.

To evaluate the diagnostic value of these 8 miRNAs in a large-scale development cohort (Table 1), the expression of the miRNAs was measured by qRT-PCR in 269 samples. Initial analysis with clustering and PCA mapping did not effectively separate patients based on clinical status (Supplementary Figure 4), and it was difficult to predict diseases by using only one miRNA (Supplementary Figure 5). Thus, to develop an optimal predictive algorithm by using multiple miRNAs, cross-validation analysis was performed as described in Supplementary Figure 6. The sample was randomly split into a CV training dataset and a CV test dataset 1,000 times, and the AUC was calculated for models based on every possible combination of the miRNAs. Equations to compute the probability of OvCa were based on logistic regression models with highest AUC values for both the training dataset and the test dataset. The best diagnostic performance values obtained for the identification of OvCa patients vs. healthy controls were 0.968 [95% confidence interval (CI), 0.948-0.989] for the AUC, 0.921 for sensitivity and 0.910 for specificity, at the optimal cut-off points (Figure 3A). With the addition of CA-125, the conventional serum biomarker for OvCa, performance further improved; the optimal model, selected after considering all possible combinations with the 8 miRNAs, included CA-125 and 6 miRNAs (miR-200a-3p, miR-766-3p, miR-26a-5p, miR-142-3p, let-7d-5p and miR-328-3p), and had an AUC of 0.994 (95% CI, 0.988-0.999) (Figure 3B), sensitivity of 0.984, and specificity 0.956 at the optimal cut-off points. The formulas derived from all the prediction models are shown in Supplementary Table 6.

**Table 1:** Characteristics of patients in validation study

Characteristic	N
Study population	269
Age, years	54.1 (mean)
BMI	25.6 (mean)
Ethnic background	
Caucasian	219
Asian	50
Ovarian cancer	155
Histopathological subtype	
Serous adenocarcinoma	112
Mucious adenocarcinoma	11
Endometrioid adenocarcinoma	13
Clear cell adenocarcinoma	19
Stage	
I	52
II	13
III	86
lV	4
Borderline tumor (LPM)	8
Histopathological subtype	
Mucious adenocarcinoma	7
Endometrioid adenocarcinoma	1
Benign diseases	43
Histopathological subtype	
Serous cystadenoma	28
Mucinous cyst adenoma	8
Endometrial cyst	7
Healthy controls	63

### Detection of early-stage cancer in patients with ovarian tumors

When tumors are incidentally detected on transvaginal ultrasonography, distinguishing benign tumors from malignant cancers is a major concern for gynecologists. Currently, it is impossible to establish a definite diagnosis without surgery, and needle biopsy is proscribed for ovarian tumors because it may result in the dissemination of cancerous cells in the peritoneal cavity. To address these limitations, we developed a predictive algorithm that differentiates early-stage cancers from benign tumors using 7 miRNAs (miR-200a-3p, miR-766-3p, miR-26a-5p, miR-142-3p, let-7d-5p, miR-130b-3p and miR-328-3p). These 7 miRNAs were selected after analyzing models featuring all possible combinations of 8 miRNAs. The diagnostic performance values of the optimal model were calculated as shown above; the AUC was 0.902 (95% CI, 0.844-0.960), the sensitivity was 0.861, and the specificity was 0.833 (Figure 3C). Furthermore additional comparison by using prediction models developed herein were performed (Supplementary Figure 7). These results suggest that the predictive model based on this combination of miRNAs is highly accurate in distinguishing patients with early-stage OvCa from healthy controls and patients with benign tumors.

### Subtype classification using the 8-miRNA signature

**Figure 4:**
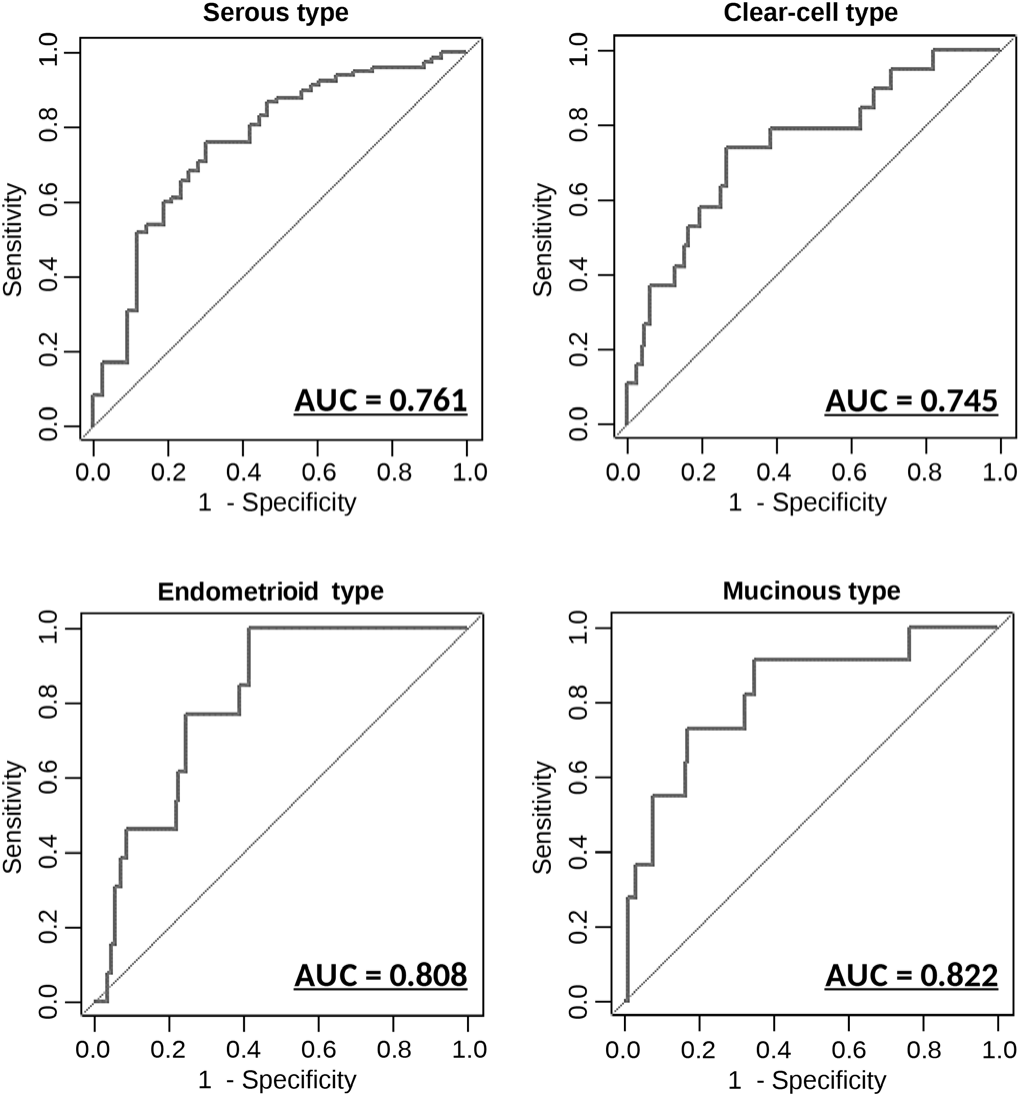
Diagnostic outcomes in each model for the prediction of histopathological subtypes ROC curves for the discrimination of each histopathological subtype versus other subtypes AUC values are shown on the graphs. N = 155 (serous: 112; mucinous: 11; Endometrioid: 13; clear-cell: 19).

Epithelial OvCa consists of the following 4 major histological subgroups: serous, clear-cell, endometrioid and mucinous, each of which has distinct molecular and pathological characteristics. Identification of subtypes in OvCa before surgery provides useful information for clinicians because drug response and prognosis differ by subtype [14]. We performed further statistical analyses using the 8 miRNAs identified earlier, to develop classification models that can distinguish different OvCa subtypes based on serum miRNA expression levels. As shown in Figure 4, the diagnostic performance values for these models were calculated in the same manner as for OvCa prediction. The 8-miRNA classification model had AUC, sensitivity and specificity values of 0.761 (95% CI, 0.674-0.849), 0.759 and 0.698, respectively, for serous OvCa, 0.745 (95% CI, 0.622-0.870), 0.737 and 0.735, respectively, for clear-cell OvCA, 0.808 (95% CI, 0.716-0.900), 0.769 and 0.754, respectively, for endometrioid OvCA, and 0.822 (95% CI, 0.683-0.961), 0.727 and 0.833, respectively, for mucinous OvCA. These findings indicate that the expression levels of circulating serum miRNAs are representative of histopathological subtypes. In other words, different types of OvCa cells may release different types and levels of miRNAs in body fluids.

### Ovarian cancer cells secrete miRNAs packaged in EVs

**Figure 5:**
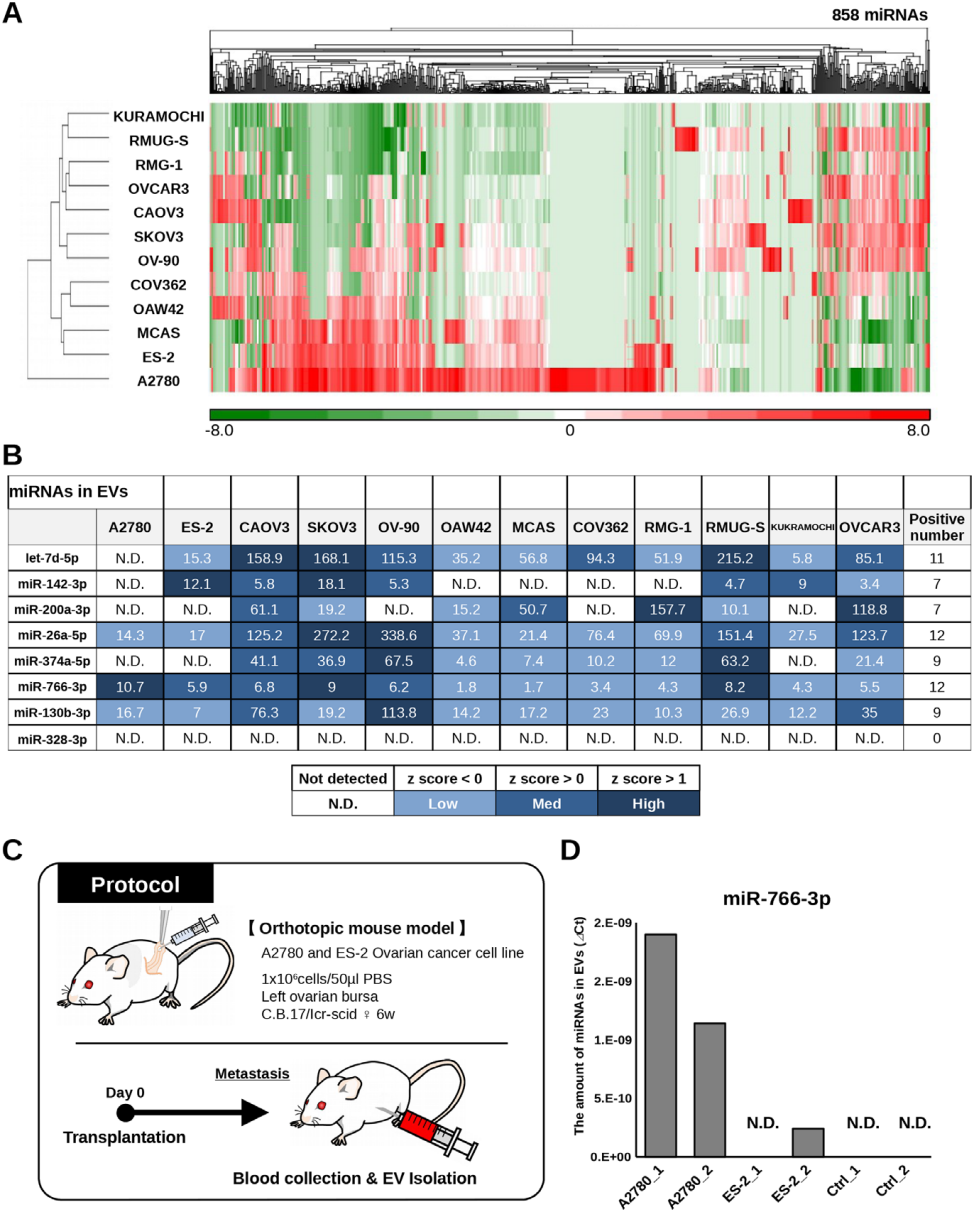
Validation of selected miRNAs in ovarian cancer cell lines **(A)** Heat map showing the levels of miRNAs in exosomes derived from ovarian cancer cell lines. **(B)** Table showing the signal intensity of 8 selected miRNAs in EVs derived from ovarian cancer cell lines in microarray analysis. Each spot is colored according to the Z-score. **(C)** Schematic of the protocol for identifying circulating miRNAs in EVs derived from ovarian cancer cells. Orthotopic mouse models were established with A2780 cells and ES-2 cells, and blood was collected from the mice on day 10 (ES-2 cells) and day 21 (A2780 cells). **(D)** Levels of miR-766-3p in mouse serum EVs as assessed by qRT-PCR. The vertical axis indicates the ⊿Ct value normalized to the levels of miR-766-3p. A2780_1 and _2: orthotopic mouse model with A2780 cells. ES-2_1 and _2: orthotopic mouse model with ES-2 cells. Ctrl_1 and _2: mouse without human ovarian cancer cells.

Almost all living cells secrete EVs, including exosomes, which are small membranous vesicles that carry small RNAs, including miRNAs [15]. Recently, EVs have attracted major interest as a potential target for new diagnostic biomarkers in various diseases [16, 17]. Although it is known that not all circulating miRNAs are packaged in EVs [18], we sought to investigate whether the 8 miRNAs we identified were associated with EVs and released from OvCa cells. Total RNA was extracted from EVs derived from the culture supernatant of 12 OvCa cell lines, and microarray analysis was performed to examine the distribution of miRNAs (Figure 5A). As shown in the heat map, 7 of the miRNAs (all except miR-328-3p) were detected in EVs derived from cancer cell lines (Figure 5B). To further confirm that the selected miRNAs detected in serum were derived from ovarian tumor epithelial cells, *in vivo* experiments were performed. Orthotopic mouse models were established by transplanting A2780 and ES-2 OvCa cell lines, and blood was collected from the mice after the establishment of early-phase metastasis (Figure 5C). Of the selected 8 miRNAs, miR-766-3p was the only one that is found in humans but not in mice. The amount of circulating miR-766-3p in EVs in the mouse serum samples was measured by qRT-PCR. EV-associated miR-766-3p was detected in mice with human OvCa, but not in those with no tumor (control 1-2). These data demonstrated that the selected miRNA could be derived from OvCa cells and that the majority of the miRNAs were packaged in EVs.

## DISCUSSION

Early detection of OvCa is particularly important for improving patient outcomes [2–4], as optimal cancer prevention is not realistically feasible given the multitude of cancer risk factors [1]. In this study, we successfully developed two types of prediction models by combining the expression levels of 8 miRNAs, and the models showed excellent diagnostic performance. The fact that the 8 miRNAs selected in this study have previously been reported as functional miRNAs in OvCa [18, 19] suggests that the selection process worked well. Among the 8 miRNAs, miR-200a is known to be involved in OvCa tumorigenesis [20], and miR-26a possesses similar functions [21]. In addition, miR-374a is reported to regulate cisplatin resistance in OvCa cells [22]. Other miRNAs are also reported as functional miRNAs that are involved in cancer pathogenesis [23–26]. The first model discriminates OvCa patients from healthy controls, assuming use in general screening; the second model distinguishes early-stage cancer from benign tumors, which is useful in routine clinical practice for gynecologists. In addition, the data presented here demonstrate the potential of miRNA panels to predict histopathological subtypes, although the size of each cohort in this study was not large enough, and independent validation would be needed to develop clinical applications. This model could provide information on tumor subtype before initiation of treatment, which would be highly valuable because subtype is tightly linked to tumor origin and can guide treatment strategies. For example, serous-type cancer cells originate from the fimbria of the fallopian tubes and could be diagnosed not only as OvCa but also as peritoneal cancer or fallopian tube cancer [27]. Furthermore, patients with clear-cell OvCa could also have endometriosis and would be poor candidates for surgical procedure [28]. Thus, the information provided by these classification models may be useful and could lead to appropriate preparation for treatment.

CA-125 has been used as the single biomarker for OvCa [29–32] in screening trials for a long time, but its specificity is limited. It is only elevated in approximately 50% of stage I cases, and approximately 80% of advanced cases [33]. Unfortunately, several recent studies using CA-125 as the single biomarker revealed no improvement in diagnostic power in pre-clinical samples [29, 34]. Thus, better biomarkers for OvCa have been needed for a long time. In this context, the use of circulating miRNAs as new biomarkers [35, 36] is a welcome development. In fact, several studies have reported the utility of circulating serum miRNAs in the past several years [37–39]. In 2009, Resnick et al. analyzed 28 serum samples from OvCa patients and 15 from healthy controls, and identified 8 miRNAs that were significantly differentially expressed by qRT-PCR [37]. In 2013, Guo et al. analyzed 50 serum samples from OvCa patients and 50 from healthy controls, and found that miR-92 levels were significantly higher in cancer patients [38]. In addition, there were significant correlations between miR-92 expression, regional lymph node involvement and clinical stage of the tumor. In the same year, Hong et al, described the potential of miR-221 as an OvCa biomarker, by analyzing 96 serum samples from OvCa patients [39]. They found that miR-221 was upregulated in cancer patients and that its serum levels were associated with prognosis. Although these reports have clearly demonstrated the validity of serum miRNAs as cancer biomarkers, most of the studies were not performed in large-scale research cohorts (less than 100 samples) and lacked assessments of diagnostic performance for possible clinical applications [4, 40, 41]. To address these issues, our study was conducted with a larger number of clinical samples. Our prediction models showed excellent diagnostic performance and have bright prospects for clinical use. Further prospective validation is currently in planning. Nevertheless, our results show that circulating miRNA may have potential as major biomarkers in OvCa.

To investigate miRNA expression, most studies use qRT-PCR, which is widely recognized as the standard method for analyzing the expression of miRNAs. This study also used qRT-PCR to validate miRNA-seq, but this method is unfit for clinical use because it is a time-consuming and complex procedure. This may be one of the reasons why miRNA biomarkers are not presently used in clinical applications. Soon, it is expected that a new device to quantify circulating miRNAs will be developed, and the clinical use of miRNAs as disease biomarkers will then proceed rapidly.

In the last part of this study, we found that most selected miRNAs were packaged in EVs and that they could be derived from OvCa cells. EVs, including exosomes, have been intensively researched in the last decade because of their increasingly recognized value as disease biomarkers [42]. The first striking report on the diagnostic potential of miRNA in EVs was published by Taylor et al. in 2008 [18]. In that study, EVs were isolated from patient serum, and miRNAs in EVs and cancer tissues were analyzed. The authors reported that the profiles of 8 miRNAs were correlated between cellular and EV-expressed miRNAs, providing strong evidence for the potential to develop “liquid biopsy” methods. Since that initial report, research involving EV-related biomarkers has accelerated. Ogata-Kawata et al. found that 7 miRNAs in EVs were significantly increased in colorectal cancer, and those signatures appeared to mirror pathological changes [43]. Huang et al. reported that miR-1290 and miR-375 in EVs are promising prognostic biomarkers for prostate cancer [44]. Chiam et al. suggested that 10 miRNAs could serve as new biomarkers for the detection of esophageal cancer [45]. EVs contain cell surface proteins as well as miRNAs and other molecules. If OvCa-specific cell surface proteins can be identified, it may be possible to capture cancer-specific EVs by ELISA or other methods [46]. Recently, research on the detection of specific EVs using new tools has also expanded [17]. EV-expressed miRNAs have great potential as biomarkers, and they could also help to elucidate disease mechanisms. In breast cancer, miR-105 and miR-181c in EVs may induce breakdown of the blood-brain barrier and promote brain metastasis [47]. Furthermore, miR-21 in EVs derived from stromal cells may influence malignant phenotypes, such as drug resistance, in metastatic OvCa cells [48]. Therefore, the fact that the miRNAs selected in the present study are expressed in EVs may be useful in generating further insights into the functions of circulating miRNAs and developing new technologies for cancer detection.

## MATERIALS AND METHODS

### Patients and sample preparation

Serum samples from preoperative patients with an adnexal mass suspicious for malignancy were collected from 2014 to 2016 at the National Cancer Center Hospital (N = 40) and Nagoya University Hospital (N = 10). An additional 284 samples from similar patients as well as healthy controls were purchased from ProteoGenex (Culver City, CA, USA). The Institutional Review Boards at the National Cancer Center (number: 2014–164) and the Nagoya University Graduate School of Medicine (number: 4881) approved this study, and all materials were obtained with written informed consent. Total RNA was extracted from the serum (200 μL for qRT-PCR and 600 μL for NGS) using QIAzol and the miRNeasy Mini Kit (Qiagen, Hilden, Germany) according to the manufacturer's protocols.

### Library preparation and Sequencing

Total RNA extracted from 600 μL of each serum sample was used to construct a sequencing library with the TruSeq Small RNA Sample Prep Kit (Illumina), as outlined by the manufacturer's protocol. Quality control of the libraries was performed using the Bioanalyzer system (Agilent). The pooled libraries created from the samples were sequenced using the Illumina HiSeq system in 51-base pair (bp) single-end reads. Before analysis of the small RNA-Seq data, the adaptor sequences were trimmed, and non-small RNA-related reads (e.g., null inserts [insert size < 15nt], long inserts [insert size > 28 nt], 5' adapter contaminants and sequences containing poly-A tails) were removed by a custom Perl script. Trimmed sequence reads were aligned to the human reference genome (hg19) using COBWeB aligner implemented in StrandNGS ver. 2.6 (Agilent Technologies, Santa Clara, CA, USA). Mismatches of 1 bp were allowed in the alignment. The read counts allocated for each small RNA were quantified using the Trimmed Mean of M-value (TMM) method [49].

### qRT-PCR

For qRT-PCR analysis, complementary DNA was generated from total RNA using a TaqMan® MicroRNA Reverse Transcription Kit (Thermo Fisher Scientific Inc.) according to the manufacturer's protocols. Real-time PCR was then performed in duplicate with a 1:4 dilution of cDNA using a Universal PCR Master Mix (Applied Biosystems). The data were collected and analyzed using StepOne Software v2.3 (Applied Biosciences). The miRNA quantification data in the final development cohort was normalized to the expression of miR-16. All TaqMan MicroRNA assays were purchased from Applied Biosystems.

### Cell lines

Human OvCa cell lines were purchased from the American Type Culture Collection (ATCC), the European Collection of Cell Cultures (ECACC) and the Japanese Collection of Research Bioresources (JCRB) cell bank. The SKOV3, OVCAR3, CAOV3, ES-2, and OC-90 lines were from the ATCC, the A2780, OAW42, and COV362 were from the ECACC, and the MCAS, RMG-1, RMUG-S, and KURAMOCHI lines were from JRCB. All cell lines were cultured in optimal medium according to the suppliers’ recommendations. Total RNA was extracted from cultured cells or extracellular vesicles (EVs) derived from cell culture supernatants using QIAzol and the miRNeasy Mini Kit (Qiagen, Hilden, Germany) as instructed by the manufacturer's protocols.

### EV purification and analysis

The cells were washed with phosphate-buffered saline (PBS), and the culture medium was replaced with advanced Dulbecco's modified Eagle's medium for ES-2, SKOV3, CAOV3, OV-90, OAW42, COV362 and MCAS cells, advanced DMEM/Ham's F-12 medium for RMG-1 and RUG-S cells, or advanced RPMI medium for A2780, OVCAR3 and KURAMOCHI cells. In all cases, the cell culture medium contained antibiotic and antifungal drugs as well as 2 mM L-glutamine (but not FBS). After incubation for 48 h, the conditioned medium (CM) was collected and centrifuged at 2,000 x *g* for 10 min at 4°C. To thoroughly remove cellular debris, the supernatant was filtered through a 0.22-μm filter (Millipore). The CM was then used for EV isolation. To prepare EVs, CM was ultracentrifuged at 35,000 rpm using a SW41Ti rotor for 70 min at 4°C. The pellets were washed with PBS, ultracentrifuged at 35,000 rpm using the SW41Ti rotor for 70 min at 4°C and resuspended in PBS.

### Microarrays

Total RNA was extracted from cultured cells using QIAzol reagent and the miRNeasy Mini Kit (Qiagen). The quantity and quality of extracted RNA were determined using a NanoDrop ND-1000 spectrophotometer (Thermo Fisher Scientific Inc.) and the Agilent Bioanalyzer system (Agilent Technologies), as recommended. Total RNA was labeled with cyanine 3 (Cy3) using the miRNA Complete Labeling and Hyb Kit (Agilent Technologies) as instructed by the manufacturer. Briefly, total RNA was dephosphorylated by incubating with Calf Intestinal Alkaline Phosphatase (CIP) Master Mix at 37°C for 30 min. Dephosphorylated RNA was denatured by incubating with DMSO at 100°C for 5 min and then immediately transferred to ice for 2 min. After addition of a ligation master mix for T4 RNA Ligase and Cyanine 3-Cytidine bisphosphate (Cy3-pCp), the RNA was incubated at 16°C for 2 h. Labeled RNA was dried using a vacuum concentrator at 55°C for 1.5 h, then hybridized onto Agilent SurePrint G3 Human miRNA 8×60K Rel.19 (design ID: 046064) arrays at 55°C for 20 h. After washing, the microarrays were scanned using an Agilent DNA microarray scanner. The intensity values for each scanned feature were quantified using Agilent Feature Extraction software version 10.7.3.1, which performs background subtractions. We only used features that were flagged as having no errors (detected flags) and excluded features that were not positive, not significant, not uniform, not above background, saturated, or population outliers (undetected flags). The expression analysis was performed with Agilent GeneSpring GX version 13.0. There were a total of 2,006 miRNA probes on the SurePrint G3 Human miRNA 8×60K Rel.19 (design ID: 046064) array, excluding the control probes.

### Animal experiments

Animal experiments were performed in compliance with the guidelines of the Institute for Laboratory Animal Research and the National Cancer Center Research Institute (Number: T14-013). Female CB-17/Icr-scid/scidJcl mice (CLEA, Tokyo, Japan) 6-7 weeks of age were used in the experiments. An orthotopic OvCa mouse model was established as previously described [50]. The IVIS Spectrum imaging system (Caliper Life Science, Hopkinton, MA) was used to verify the onset of peritoneal metastasis. The mice were administered 150 mg/kg D-luciferin (Promega, Madison, WI) by intraperitoneal injection. Ten minutes later, photons in the whole bodies of the animals were measured by assessing bioluminescence. After cancer cells had metastasized, blood was obtained by cardiac puncture. After generating serum from the blood, EVs were isolated using Exosome Isolation Reagent (Thermo Fisher Scientific Inc) according to the manufacturer's instructions.

### Statistical analysis

To identify the optimal combination of miRNAs for prediction, we used the miRNA dataset normalized with miR-16 and the training-validation strategy displayed in Supplementary Figure 6. First, we randomly split the data into a training dataset and a test dataset, for two-fold cross-validation (CV). Using the training dataset, we developed prediction models based on logistic regression with the best subset selection method. The eight miRNAs that we identified in the first step of the study generated 255 (= _8_C_1_ + _8_C_2_ + … + _8_C_8_) possible prediction models. When CA-125 was added as a variable, 511 (= _9_C_1_ + _9_C_2_ + … + _9_C_9_) models were generated. For each model, the area under the curve (AUC) of the receiver-operating characteristic (ROC) curve for the training and test datasets were calculated. This two-fold CV step was repeated 1,000 times and the 1,000 AUCs for the training and test datasets were averaged for each model. The top 10 prediction models, which all had an average AUC greater than 0.7 for both datasets, were selected. All statistical tests were two-sided. All the analyses were performed using SAS software (version 9.3, SAS Institute Inc., Cary, NC, USA).

## SUPPLEMENTARY MATERIALS FIGURES AND TABLES






