# Supplementary material for: A combination of circulating miRNAs for the early detection of ovarian cancer
Source: Oncotarget. 2017 Sep 6;8(52):89811–23. doi: 10.18632/oncotarget.20688 (PMC5685711; doi:10.18632/oncotarget.20688)
Supplement: Supplementary file 1 [file oncotarget-08-89811-s001.pdf]

A combination of circulating miRNAs for the early detection of ovarian cancer

SUPPLEMENTARY MATERIALS

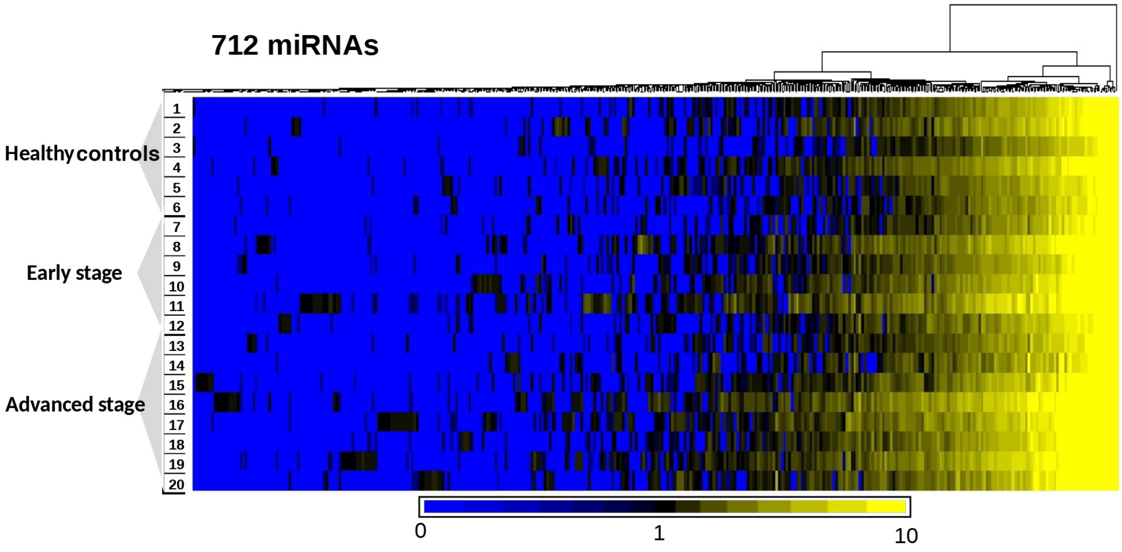

Supplementary Figure 1: Heat map of detected miRNAs in miRNA-seq. The heat map shows the distribution of read counts for 712 miRNAs that were detected by miRNA-seq. The data shown were obtained with no mismatch allowed.

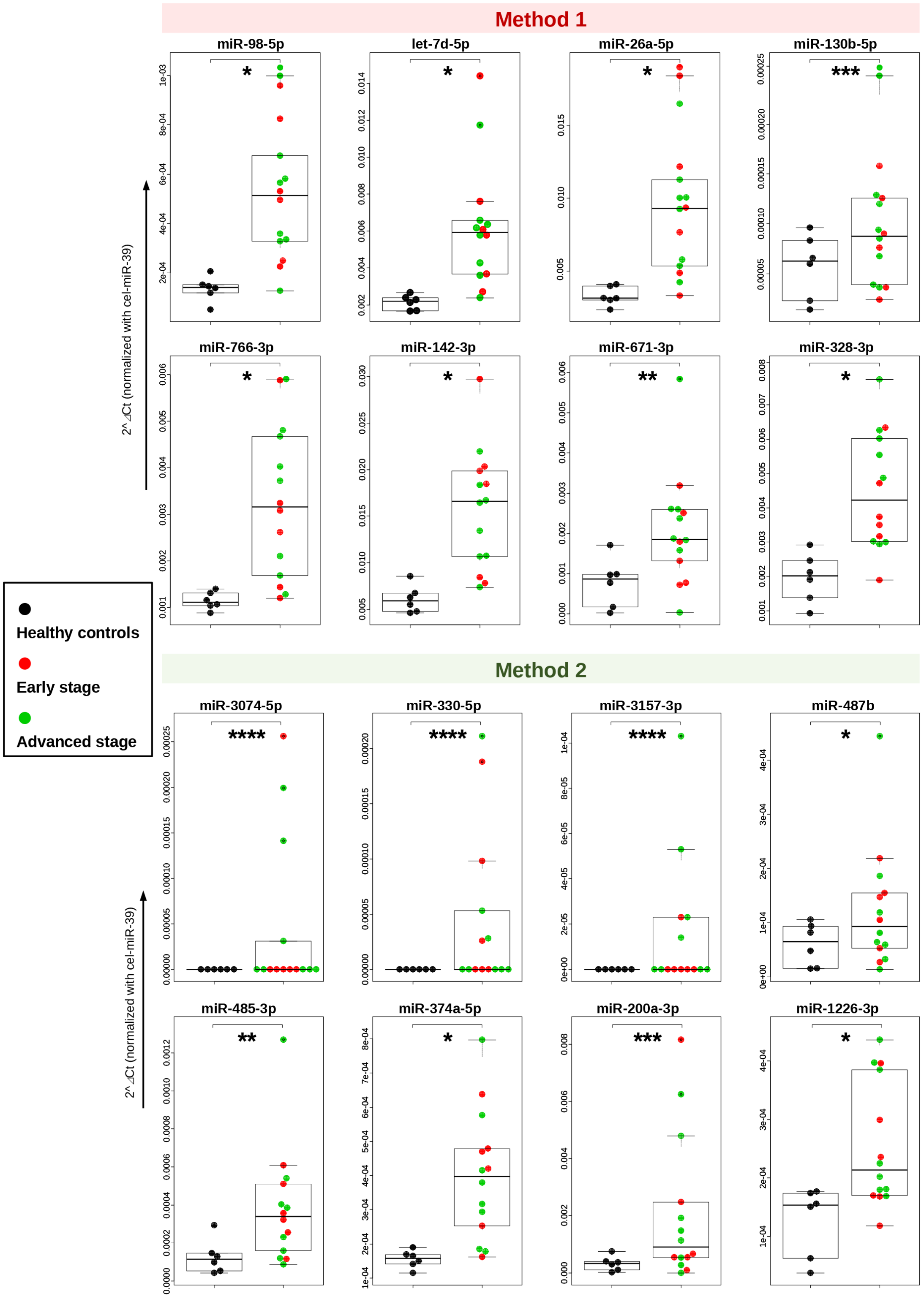

Supplementary Figure 2: Distributions of 29 selected miRNAs in the discovery cohort. Serum levels of miRNAs were measured by qRT-PCR. The dot plots are overlaid with box plots. The vertical axis shows 2^⊿Ct values, which were normalized to the levels of cel-miR-39. Descriptions of the data points are shown to the left of the graphs. The Mann–Whitney *U*-test was used. *p < 0.01, **p < 0.05, ***No significance, **** Results according to the all-or-none principle.

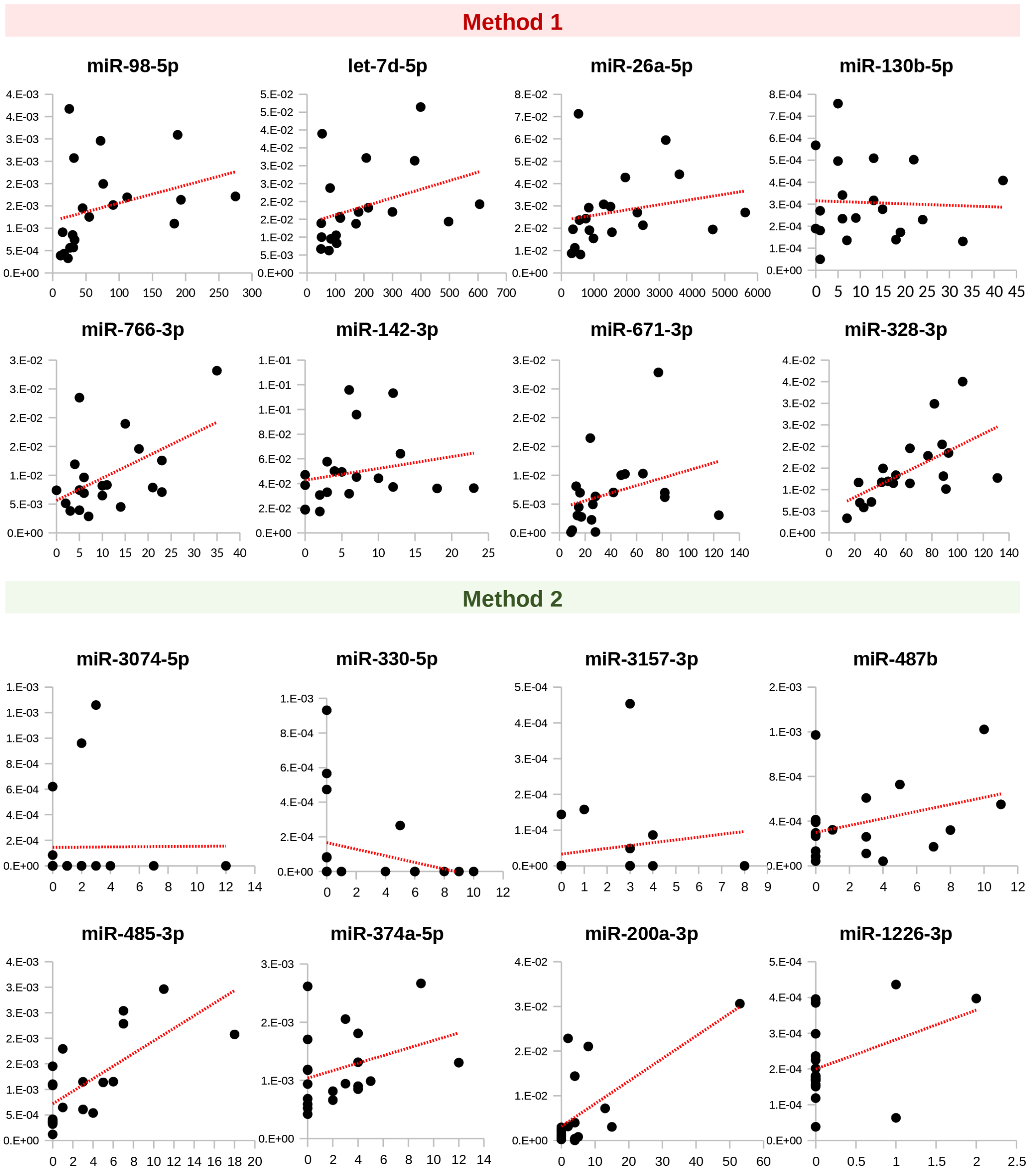

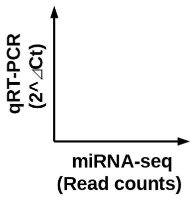

Method 1

microRNA	correlation coefficient
miR-26a-5p	0.220
miR-328	0.606
miR-671-3p	0.323
miR-98	0.309
let-7d-5p	0.355
miR-130b-5p	-0.044
miR-142-3p	0.212
miR-766-3p	0.518

Method 2

microRNA	correlation coefficient
miR-1226-3p	0.389
miR-200a-3p	0.681
miR-3074-5p	0.007
miR-3157-3p	0.160
miR-330-5p	-0.268
miR-374a-5p	0.326
miR-485-3p	0.705
miR-487b	0.334

Supplementary Figure 3: Correlations between miRNA-seq and qRT-PCR in selected miRNAs. The scatter plots of correlations between miRNA-seq and qRT-PCR. All miRNAs were selected in validation 1. Read counts are on the horizontal axis, and values of 2^⊿Ct are on the vertical axis. The values of correlation coefficient were provided in the bottom of the figure.

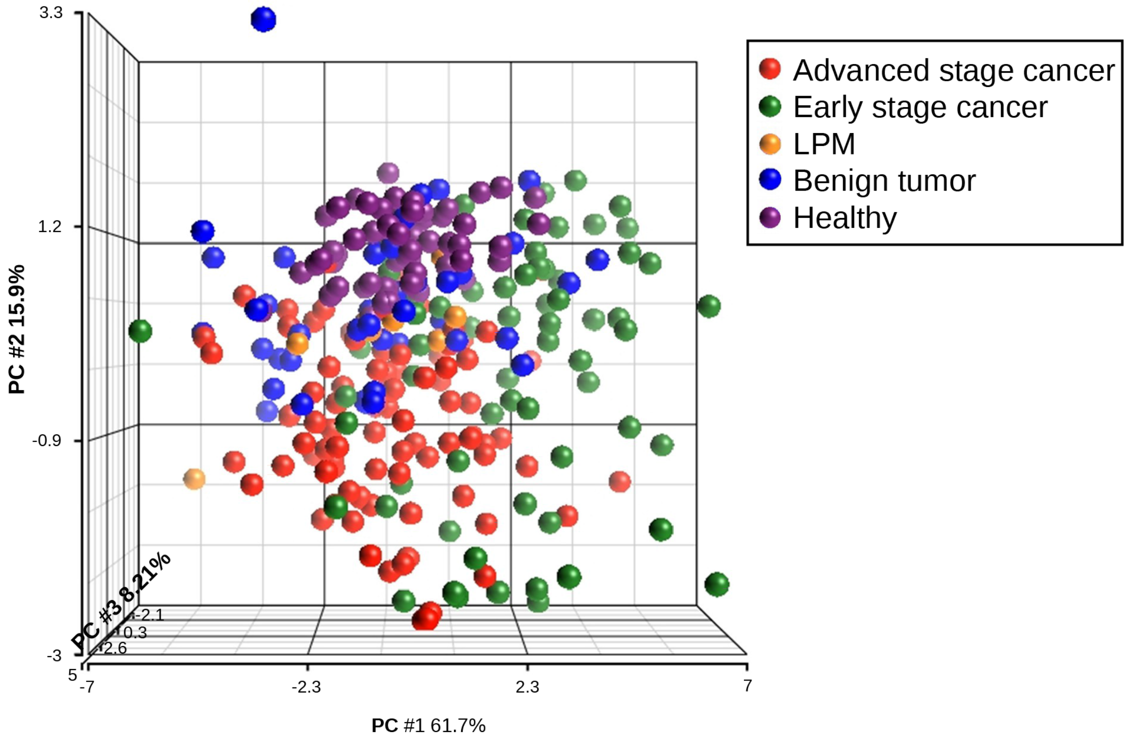

Supplementary Figure 4: RT-PCR results in the development cohort. PCA mapping of the RT-PCR results in the development cohort. Levels of 8 miRNAs and CA-125 were analyzed in 269 samples. All data for each background are represented by different colors.

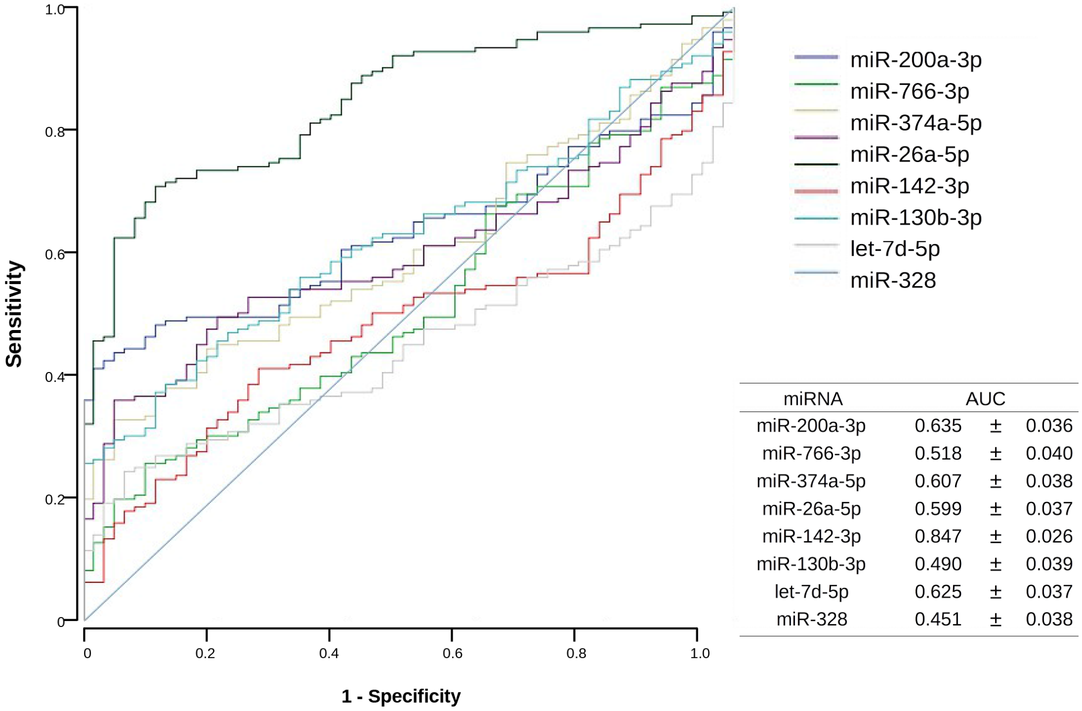

miRNA	AUC		
miR-200a-3p	0.635	±	0.036
miR-766-3p	0.518	±	0.040
miR-374a-5p	0.607	±	0.038
miR-26a-5p	0.599	±	0.037
miR-142-3p	0.847	±	0.026
miR-130b-3p	0.490	±	0.039
let-7d-5p	0.625	±	0.037
miR-328	0.451	±	0.038

Supplementary Figure 5: Diagnostic outcomes of each miRNA for predicting ovarian cancer. A ROC curve for the identification of patients with ovarian cancer (N = 155) versus healthy controls (N = 63) using 8 miRNAs.

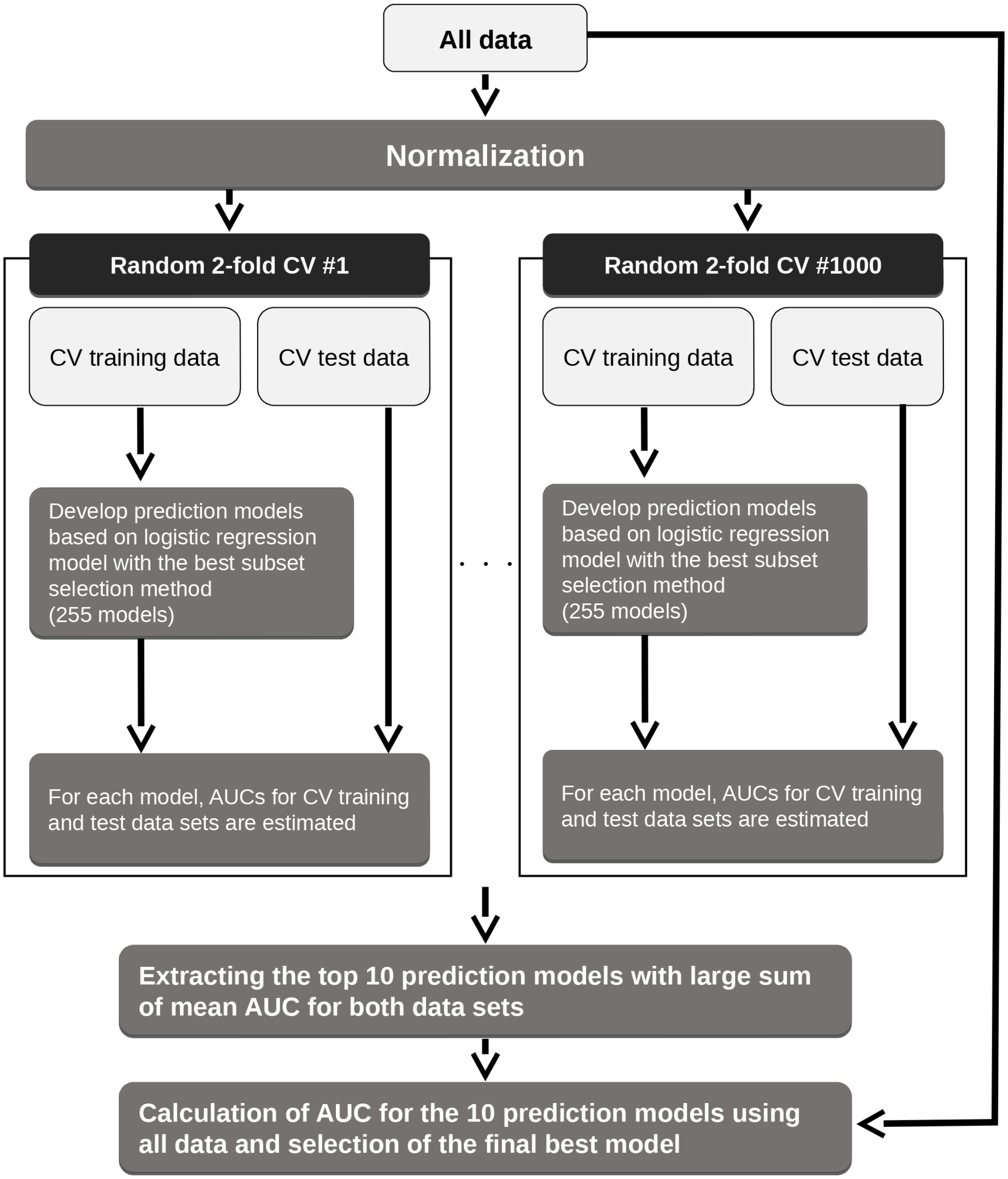

Supplementary Figure 6: Prediction model development. Overview of the statistical cross-validation method. A detailed explanation is included in the Methods section. CV: cross-validation.

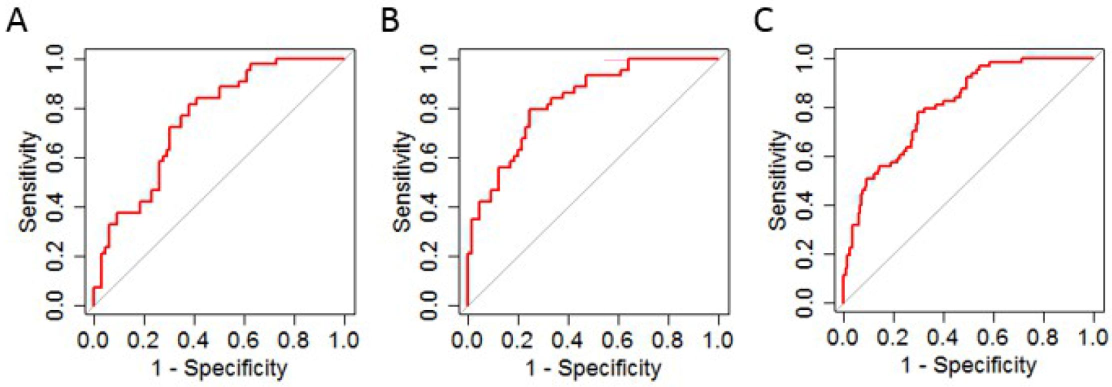

ROC	Model	Distinction	AUC
A	Fig 3A	Benign tumor vs. Early cancer	0.7551
B	Fig 3B	Benign tumor vs. Early cancer	0.8298
C	Fig 3C	Healthy controls vs. Cancer patients	0.8087

Supplementary Figure 7: Diagnostic outcomes of each models. **(A)** A ROC curve for the identification of patients with a benign tumor (N = 43) versus patients with early-stage ovarian cancer (N = 66) using the model shown in Figure 3A. **(B)** A ROC curve for the identification of patients with a benign tumor (N = 43) versus patients with early-stage ovarian cancer (N = 66) using the model shown in Figure 3B. **(C)** A ROC curve for the identification of patients with ovarian cancer (N = 155) versus healthy controls (N = 63) using the model shown in Fig. 3C. Details, including AUC values, are shown under the graphs as a table

Supplementary Table 1: The sample list for global analysis

Sample Number	Age	Sex	Ethnic background	BMI	Histological diagnosis	Stage	Grade	TNM
1	55	Female	Caucasian	26.1	N/A	N/A	N/A	N/A
2	59	Female	Caucasian	26.9	N/A	N/A	N/A	N/A
3	56	Female	Caucasian	25.8	N/A	N/A	N/A	N/A
4	50	Female	Caucasian	27.7	N/A	N/A	N/A	N/A
5	57	Female	Caucasian	28.4	N/A	N/A	N/A	N/A
6	57	Female	Caucasian	24.7	N/A	N/A	N/A	N/A
7	17	Female	Caucasian	18.0	serous adenocarcinoma	IB	N/A	T1bN0M0
8	43	Female	Caucasian	24.2	serous adenocarcinoma	IA	G1	T1aN0M0
9	63	Female	Caucasian	27.5	serous adenocarcinoma	IA	N/A	T1aN0M0
10	38	Female	Caucasian	26.3	serous adenocarcinoma	IC2	G3	T1cN0M0
11	23	Female	Caucasian	21.1	mucinous adenocarcinoma	IA	N/A	T1aN0M0
12	49	Female	Caucasian	28.1	endometrioid adenocarcinoma	IC	G3	T1cN0M0
13	58	Female	Caucasian	25.8	serous adenocarcinoma	IIIC	G2	T3cN0M0
14	28	Female	Caucasian	18.1	serous adenocarcinoma	IIIA	N/A	T3aN0M0
15	40	Female	Caucasian	21.7	serous adenocarcinoma	IIIA	N/A	T3aN0M0
16	46	Female	Caucasian	24.6	serous adenocarcinoma	IV	G3	T3bN0M1
17	39	Female	Caucasian	22.0	serous adenocarcinoma	IIIC	G3	T3cN0M0
18	64	Female	Caucasian	25.1	mucinous adenocarcinoma	IIIC	G1-2	T3cN0M0
19	57	Female	Caucasian	35.0	mucinous adenocarcinoma	IIIB	G1	T3bN0M0
20	73	Female	Caucasian	29.0	serous adenocarcinoma	IIIC	G3	T3cN0M0

N/A: nothing applicable

Supplementary Table 2: Summary of sequence reads and expression in serum samples

	Input reads		5' adaptor contaminating		Reads having 3' adaptors		Insert null		Smaller than 10nt		poly A containing		Survived reads	
Sample	Count	Rate	Count	Rate	Count	Rate	Count	Rate	Count	Rate	Count	Rate	Count	Rate
1	16668915	100.00%	20625	0.12%	14920797	89.51%	101062	0.61%	6038584	36.23%	1407	0.01%	10507237	63.03%
2	21853182	100.00%	326882	1.50%	19027998	87.07%	2343605	10.72%	4772102	21.84%	1081	0.00%	14409512	65.94%
3	14298667	100.00%	183533	1.28%	12392183	86.60%	1223216	8.55%	4676224	32.70%	155	0.00%	8215539	57.46%
4	14568492	100.00%	12590	0.09%	13036261	89.48%	49659	0.34%	5486803	37.66%	1201	0.01%	9018239	61.90%
5	16628074	100.00%	21464	0.13%	14983416	90.11%	122436	0.74%	5623543	33.82%	432	0.00%	10860199	65.31%
6	18590463	100.00%	524785	2.82%	15481164	83.27%	3676069	19.77%	4326434	23.27%	935	0.01%	10062240	54.13%
7	15464674	100.00%	737204	4.77%	12236022	79.12%	5090154	32.91%	2213472	14.31%	26	0.00%	7423818	48.01%
8	17054776	100.00%	43309	0.25%	14476499	84.88%	310285	1.82%	3866558	22.67%	191	0.00%	12834433	75.25%
9	14219489	100.00%	299843	2.11%	12026236	84.58%	2421218	17.03%	2625829	18.47%	23	0.00%	8872576	62.40%
10	12184689	100.00%	49280	0.40%	10500421	86.18%	416734	3.42%	979151	8.04%	343	0.00%	10739181	88.14%
11	13095010	100.00%	130314	1.00%	11194822	85.49%	882265	6.74%	2033436	15.53%	1257	0.01%	10047738	76.73%
12	12802308	100.00%	292397	2.28%	10690820	83.51%	2291375	17.90%	1967963	15.37%	71	0.00%	8250502	64.45%
13	14377747	100.00%	336110	2.34%	12113337	84.25%	2407675	16.75%	2012890	14.00%	86	0.00%	9620986	66.92%
14	17803472	100.00%	289476	1.63%	15370822	86.34%	2753292	15.46%	5169145	29.03%	73	0.00%	9591486	53.87%
15	12608500	100.00%	480992	3.81%	9922180	78.69%	3802899	30.16%	544236	4.32%	150	0.00%	7780223	61.71%
16	15591164	100.00%	13973	0.09%	13380308	85.82%	196346	1.26%	721990	4.63%	131	0.00%	14658724	94.02%
17	11873415	100.00%	9672	0.08%	10641883	89.63%	175589	1.48%	1019350	8.59%	48	0.00%	10668756	89.85%
18	11966233	100.00%	8587	0.07%	10499499	87.74%	127132	1.06%	1066593	8.91%	98	0.00%	10763823	89.95%
19	13725243	100.00%	12800	0.09%	11169265	81.38%	180580	1.32%	1056237	7.70%	492	0.00%	12475134	90.89%
20	12152731	100.00%	14696	0.12%	9814261	80.76%	432919	3.56%	706308	5.81%	1226	0.01%	10997582	90.49%

Supplementary Table 3: miRNA-seq data with no mismatch allowed.

See Supplementary File 1

Supplementary Table 4: miRNA-seq data with 1-bp mismatch allowed.

See Supplementary File 2

Supplementary Table 5: Sample list for analyses in the trial cohort

Sample Number	Age	Sex	Ethnic background	BMI	Histological diagnosis	Stage	Grade	TNM
1	63	Female	Caucasian	28.7	N/A	N/A	N/A	N/A
2	60	Female	Caucasian	25.5	N/A	N/A	N/A	N/A
3	53	Female	Caucasian	25.8	N/A	N/A	N/A	N/A
4	70	Female	Caucasian	24.4	N/A	N/A	N/A	N/A
5	40	Female	Caucasian	26.7	N/A	N/A	N/A	N/A
6	70	Female	Caucasian	28.6	N/A	N/A	N/A	N/A
7	75	Female	Caucasian	26.8	N/A	N/A	N/A	N/A
8	65	Female	Caucasian	27.0	N/A	N/A	N/A	N/A
9	65	Female	Caucasian	26.9	N/A	N/A	N/A	N/A
10	56	Female	Caucasian	24.9	N/A	N/A	N/A	N/A
11	60	Female	Caucasian	27.1	N/A	N/A	N/A	N/A
12	56	Female	Caucasian	25.4	N/A	N/A	N/A	N/A
13	60	Female	Caucasian	26.7	N/A	N/A	N/A	N/A
14	70	Female	Caucasian	29.3	N/A	N/A	N/A	N/A
15	65	Female	Caucasian	28.0	N/A	N/A	N/A	N/A
16	43	Female	Caucasian	24.2	serous adenocarcinoma	IA	G1	T1aN0M0
17	65	Female	Caucasian	41.4	serous adenocarcinoma	IA	G3	T1aN0M0
18	32	Female	Caucasian	19.2	serous adenocarcinoma	IA	G1	T1aN0M0
19	38	Female	Caucasian	18.4	mucinous adenocarcinoma	IA	N/A	T1aN0M0
20	18	Female	Caucasian	22.2	mucinous adenocarcinoma	IA	N/A	T1aN0M0
21	68	Female	Caucasian	19.1	mucinous cystadenocarcinoma	IA	N/A	T1aN0M0
22	56	Female	Caucasian	21.6	endometrioid adenocarcinoma	IA	N/A	T1aN0M0
23	36	Female	Caucasian	22.9	endometrioid adenocarcinoma	IA	N/A	T1aN0M0
24	54	Female	Caucasian	25.2	serous adenocarcinoma	IC	G1	T1cN0M0
25	49	Female	Caucasian	28.1	endometrioid adenocarcinoma	IC	G3	T1cN0M0
26	43	Female	Caucasian	25.4	serous adenocarcinoma	IIB	N/A	T2bN0M0
27	51	Female	Caucasian	23.5	serous adenocarcinoma	IIB	G3	T2bN0M0
28	57	Female	Caucasian	26.8	serous adenocarcinoma	IIA	G1-2	T2aN0M0
29	44	Female	Caucasian	23.7	serous adenocarcinoma	IIB	G1	T2bN0M0
30	47	Female	Caucasian	24.2	mucinous adenocarcinoma	IIC	G1	T2cN0M0
31	41	Female	Caucasian	20.7	serous adenocarcinoma	IIIA	G3	T3aN0M0
32	41	Female	Caucasian	27.6	serous adenocarcinoma	IIIB	G1-3	T3bN0M0
33	45	Female	Caucasian	24.6	serous adenocarcinoma	IIIB	G2	T3bN0M0
34	38	Female	Caucasian	39.8	serous adenocarcinoma	IIIC	N/A	T3cN0M0
35	66	Female	Caucasian	27.5	serous adenocarcinoma	IIIC	N/A	T3cN0M0
36	59	Female	Caucasian	37.7	serous adenocarcinoma	IIIC	G3	T3cN0M0
37	61	Female	Caucasian	27.9	serous adenocarcinoma	IIIC	G2	T3cN0M0
38	64	Female	Caucasian	31.6	serous adenocarcinoma	IIIC	G2	T3cN0M0
39	66	Female	Caucasian	28.0	serous adenocarcinoma	IIIC	G2	T3cN0M0
40	56	Female	Caucasian	39.1	serous adenocarcinoma	IIIC	G3	T3cN0M0
41	66	Female	Caucasian	46.1	serous adenocarcinoma	IIIC	G2	T3cN0M0
42	57	Female	Caucasian	29.7	serous adenocarcinoma	IIIC	G2-3	T3cN0M0
43	49	Female	Caucasian	23.1	serous adenocarcinoma	IIIC	G2	T3cN0M0
44	51	Female	Caucasian	38.9	serous adenocarcinoma	IIIC	N/A	T3cN0M0
45	57	Female	Caucasian	32.3	serous adenocarcinoma	IIIC	G2	T3cN0M0

N/A: nothing applicable.

Supplementary Table 6: List of prediction models expressed as formulas

The probabilities (*p*) for identification	ROC curves		Calculation formulae, *p* = exp(a) / { 1 + exp(a)}A
Cancers from healthy	Figure 3A	a =	(3.4) + (-2346.1) x miR-200a-3p + (245.2) x miR-766-3p + (2706.2) x miR-374-5p + (512.5) x miR-26a-5p + (-269.4) x miR-142-3p + (-2575.7) x miR-130b-3p + (-634.8) x let-7d-5p + (0.3) x miR-328-3p
Cancers from healthy	Figure 3B	a =	(6.5) + (-1767.6) x miR-200a-3p + (236.0) x miR-766-3p + (495.5) x miR-26a-5p + (-163.5) x miR-142-3p + (-522.4) x let-7d-5p + (-68.9) x miR-328-3p + (-0.2) x CA-125
Early cancers from benign tumors	Figure 3C	a =	(3.0) + (-247.3) x miR-200a-3p + (274.5) x miR-766-3p + (-8.8) x miR-26a-5p + (-66.2) x miR-142-3p + (-1983.2) x miR-130b-3p + (24.6) x let-7d-5p + (-219.5) x miR-328-3p
Serous types from other subtypes	Figure 4	a =	(1.2)+ (0.1) x CA-125+ (-50.4) x miR-766-3p + (-698.6) x miR-374-5p + (23.7) x miR-142-3p + (3.6) x let-7d-5p + (11.9) x miR-328-3p
Clear-cell types from other subtypes	Figure 4	a =	(-1.4) + (-88.8) x miR-374 + (14.7) x miR-26a-5p + (-21.9) x miR-142-3p + (1686.1) x miR-130b-3p + (48.0) x let-7d-5p + (-78.4) x miR-328-3p + (-0.1) x CA-125
Endometrioid types from other subtypes	Figure 4	a =	(-2.6) + (-18.4) x miR-766-3p + (1056.5) x miR-130b-3p
Mucinous types from other subtypes	Figure 4	a =	(-2.4) + (22.1) x miR-200a-3p + (37.1) x let-7d-5p + (-31.2) x miR-328-3p + (-0.1) x CA-125
